# A Pilot Study to Evaluate an International Normalized Ratio-Derived Formula in Combination with Heparin-Calibrated Anti-Xa Activity in Calculating a Plasma Edoxaban Level

**DOI:** 10.3390/jcm14031006

**Published:** 2025-02-05

**Authors:** Chun-Fung Sin, Pui-Yee Chan, Yi-Teng Hoo, Wang-Ho Yuen, Hoi-Ching Wong

**Affiliations:** 1 Department of Pathology, School of Clinical Medicine, The University of Hong Kong, Hong Kong SAR, China; 2 Division of Haematology, Department of Pathology, Queen Mary Hospital, Hong Kong SAR, China

**Keywords:** international normalized ratio, plasma level, anti-Xa, heparin-calibrated anti-Xa, edoxaban level

## Abstract

**Introduction:** A drug-specific chromogenic assay is not immediately available, so it hampers the treatment of patients who present in a clinical emergency. In this pilot study, we aimed to create a formula to predict a plasma edoxaban level based on the international normalized ratio (INR) and heparin-calibrated anti-Xa activity and derive a novel workflow for routine laboratory diagnosis. **Method:** Forty-two patients prescribed edoxaban were recruited and randomized to a testing or validation cohort. Plasma levels from the testing cohort were used to create a prediction formula that was then validated in a validation cohort and real-world clinical requests. **Results:** The INR-derived formula had high sensitivity (95.8–100%) to predict the plasma edoxaban level > 50 ng/mL and >100 ng/mL but with low specificity. However, the specificity of predicting the plasma edoxaban level of ≥100 ng/mL was 100% by using an INR ≥ 1.5 as cut-off. Heparin-calibrated anti-Xa-derived formula had a high sensitivity (90.9–100%) and specificity (93.8–100%) in real clinical situations. A two-tier approach of combining INR-derived and heparin-calibrated anti-Xa-derived formulae can overcome the low specificity and utilize the advantages of wide availability and a short turnaround time of the INR-derived formula. **Conclusions:** Both INR-derived and heparin-calibrated anti-Xa-derived formulae can be applied to calculate the plasma edoxaban level. A two-tier workflow of combining these two formulae greatly helps streamline the treatment of patients prescribed edoxaban who present in a clinical emergency. Adoption of this framework is feasible for routine diagnostic laboratories.

## 1. Introduction

Atrial fibrillation (AF) is a common cardiac arrhythmia worldwide, and its incidence increases with age. Cardioembolic stroke is one of the complications of AF, and it causes significant morbidity and mortality. Recent guideline suggests DOACs as first-line treatment for stroke prophylaxis in AF patients [1]. Edoxaban is a direct anti-Xa inhibitor, and renal excretion constitutes 50% of the drug metabolism of edoxaban [2,3,4].

Dose titration according to the plasma edoxaban level is not standard practice, although there are clinical situations in which knowledge of plasma edoxaban levels would help clinical management [3]. The drug level is critical for determining the need for antidotes when presenting with bleeding complications or before invasive procedures [5,6]. Assessing plasma DOAC levels is also critical to determine whether it is safe to administer thrombolytic therapy to patients presenting with ischemic stroke [5,7]. Most importantly, there is a limited time window for administering thrombolytic therapy to such patients to achieve a reasonable risk–benefit ratio [8]. Thus, a rapid turnaround time (TAT) of the plasma DOAC level is required in those clinical situations where the plasma level must be measured urgently to guide clinical decisions. Previous studies showed that the plasma DOAC level increased with worsening renal function and were associated with an increased risk of bleeding complications [9,10]. Consequently, in patients with impaired renal excretion and a worsening renal function, the accumulation of edoxaban may render the patient prone to bleeding complications [4,11]. Plasma DOAC assays may help to optimize drug dosage in patients with renal impairment [6].

The chromogenic anti-Xa assay with edoxaban-specific calibrators is a common means to measure the plasma edoxaban level with satisfactory accuracy and precision [12]. However, an assay that is specific to edoxaban is not widely available. The low volume of the test and the necessity of setting up different methodologies and calibrators for different direct anti-Xa inhibitors impose a high cost and manpower to conduct such tests in a routine diagnostic laboratory [13]. In addition, time is needed to set up the calibration curve and perform quality control procedures, so a rapid TAT is difficult to achieve [14].

The international normalized ratio (INR), which is calibrated against different PT reagents, is a readily available coagulation screening test with a short TAT. Previous study showed the utility of the INR in predicting the plasma rivaroxaban level and facilitates clinical management [15]. Nevertheless, the change in the INR in response to the increasing plasma edoxaban level varies with different PT reagents having different international sensitivity indexes (ISIs) [16]. Moreover, various medical conditions such as liver disease and disseminated intravascular coagulation could affect the value of the INR. In these scenarios, the INR is not an ideal parameter to predict a plasma edoxaban level.

The universal anti-Xa assay for all direct Xa inhibitors using heparin-specific calibrators is the method of choice. Results have shown high concordance between the plasma edoxaban level measured by liquid chromatography–mass spectrometry (LC–MS) and the heparin-calibrated anti-Xa assay [17]. Given the wide variety of direct Xa inhibitors in use, setting up an anti-Xa assay for individual direct Xa inhibitors with drug-specific calibrators requires an extra cost and manpower. A universal anti-Xa assay with heparin-specific calibrators is an attractive approach with a lower cost and more rapid turnaround time than the specific chromogenic anti-Xa assay [13].

We conducted a pilot study of the use of the INR as a “screening” method to predict those patients whose plasma edoxaban levels exceeded the cut-off value for treatment decision, followed by further validation by an anti-Xa assay using heparin-specific calibrators. The findings of our study provide evidence that this innovative approach can facilitate clinical decision-making in an emergency. This novel two-tier workflow is advantageous for manpower saving and speeding up the turnaround time, particularly in poorly resourced areas. The principle of this approach can theoretically be applied to other direct Xa inhibitors and help streamline laboratory support in measuring plasma DOAC levels in clinical emergencies.

## 2. Methods

### 2.1. Patient Recruitment

Data were obtained from the study registered on Chinese Clinical Trial Registry (Registration Number: ChiCTR2300075841). This prospective study was approved by the Institutional Review Board of the University of Hong Kong/Hospital Authority Hong Kong West Cluster (IRB HKU/HAHKWC) in January 2022 (Reference number: UW 22-086), and informed consent was signed by all patients participating in this study. We recruited consecutive patients from the cardiology outpatient clinic of the Department of Medicine at Queen Mary Hospital. This study included patients with AF prescribed a stable dose of edoxaban for stroke prophylaxis. Patients were randomly assigned to a testing cohort (for the derivation of a prediction formula from the INR and heparin-calibrated anti-Xa assays) or a validation cohort (for the validation of the prediction formula) in a 1:1 ratio. Patients were prescribed either edoxaban 30 mg or 60 mg daily according to the dose-reduction criteria of the package insert. Those patients recruited had been prescribed edoxaban for at least 1 week (more than 5 half-lives) to ensure a stable plasma level. Patients were excluded if they 1. were non-compliant with medication; 2. were unable to provide informed consent; 3. had a history of liver disease; or 4. had evidence of concomitant coagulopathy. We defined non-compliance as less than 90% compliance with edoxaban during the study period. Drug compliance was assessed by pill-counting by nurses in the outpatient clinic. The study period was from 1 January 2022 to 31 December 2022.

### 2.2. Data Retrieval

The following baseline information was recorded for all participants: age, indication and dosage of edoxaban, body weight, liver biochemistry results for liver function testing, serum creatinine, creatine clearance (CrCl) from Cockcroft–Gault equation, and any history of medical co-morbidities including hypertension, diabetes mellitus, hyperlipidemia, ischemic heart disease, and cerebrovascular accident (CVA). The investigators responsible for data retrieval were not aware of the results of the subsequent data analysis. The following equation was used to calculate CrCl: (140 − age) × body weight/serum creatinine level × 72 (× 0.85 if female) [18].

### 2.3. Blood Sampling and Processing

The peak plasma edoxaban level was measured 2 h following ingestion and the trough level immediately prior to the next dose. Blood sampling and processing were performed according to the protocol described elsewhere [19]. Briefly, a whole-blood sample was collected in a vacuum plastic tube containing 3.2% trisodium citrate. The citrated blood sample was then centrifuged for 10 min at 3750 rpm to obtain platelet-poor plasma for further testing.

### 2.4. Coagulation Screening Tests and Plasma Edoxaban Level Assay

The laboratory staff who performed the laboratory tests were not aware of the clinical information of the recruited patients and the results of subsequent analysis. All blood samples for peak and trough plasma edoxaban levels were subjected to the measurement of PT and APTT and calculation of the INR. The PT reagent Thromorel S reagent (Siemens Healthineers, Erlangen, Germany) and the APTT reagent Actin FSL Activated PTT reagent (Siemens Healthineers) were used to measure PT and APTT, respectively. All samples were analyzed by a Sysmex CS5100 analyzer (Siemens Healthineers). We determined the INR value from PT measurement from certified plasma (Siemens Healthineers) according to a method published elsewhere [20].

Measurement of the plasma edoxaban level was performed as described previously [16]. The assay was based on the principle of the chromogenic anti-Xa assay. Briefly, a BIOPHEN DiXal kit (Hyphen BioMed, Neuville-sur-Oise, France) was used for plasma edoxaban measurement, and the assay was performed using a Sysmex CS5100 analyzer as per the manufacturer’s instructions. The plasma edoxaban level was expressed as ng/mL. We conducted a quality control procedure by using quality control materials with known edoxaban levels provided by the manufacturer, performed prior to measurement of the patients’ samples. The quality control of the assay was considered satisfactory if the edoxaban level was within the permitted limit as defined by the manufacturer. The precision of the edoxaban assay was 4. 92% of coefficient of variation (CV) at the plasma level of 25 ng/mL and 2.07% of CV at the plasma level of 150 ng/mL. The limit of detection (LOD) of the assay is 7 ng/mL.

### 2.5. Heparin-Calibrated Anti-Xa Assay

The heparin-calibrated anti-Xa activity was determined by using the kit (Stachrom heparin, Stago, Parsippany, NJ, USA), and the test was performed with a Sysmex CS-5100 analyzer according to the instruction on the package insert. It utilized the principle of the chromogenic anti-Xa assay by using heparin-specific calibrators (STA Hepanorm HBPM, Stago). The quality control of the test was passed if the results obtained from the quality control materials were within the pre-defined range as stated by the manufacturer. The plasma anti-Xa activity was expressed as IU/mL.

### 2.6. Statistical Analysis

Continuous variables are presented as mean ± standard deviation (S.D.) or median (IQR) as appropriate. Categorical data are presented as percentage and frequencies. The differences in continuous variables between the testing and validation cohorts were analyzed by Student’s *t*-test (for the parametric test) or Wilcoxon signed-rank test (for the non-parametric test) as appropriate. The differences between categorical variables were calculated by the chi-square test. Kolmogorov–Smirnov goodness-of-fit test was used to determine the normality of data.

Both peak and trough plasma edoxaban levels were used to determine the relationship between PT, APTT, INR, and heparin-calibrated anti-Xa activity and to generate and validate the prediction formula. The relationship between the plasma edoxaban level and PT, APTT, INR, and heparin-specific anti-Xa activity was analyzed by Spearman correlation. The prediction formula for the plasma edoxaban level based on the INR and heparin-specific anti-Xa activity was generated from the testing cohort by curve estimation function of computer software using linear, logarithmic, inverse, quadratic, power, compound, s-curve, cubic, power, compound, s-curve, growth, and exponential models through the principle of regression analysis. The model with the largest R square value was chosen to generate the prediction formula. The formula was validated by the Bland–Altman plot after calculating the difference between the measured and calculated plasma level. Further validation of the formula was performed in the validation cohort by the Bland–Altman plot also.

The root mean square error (RMSE) was used to calculate the error between measured plasma DOAC level and that calculated by the prediction formula. The sensitivity and specificity were calculated using the following formulae:

Sensitivity = (true positive)/(true positive + false negative) × 100%

Specificity = (true negative)/(true negative + false positive) × 100%

All statistical analyses and curve estimation were performed using IBM SPSS software version 27, and a *p*-value < 0.05 was considered statistically significant.

## 3. Results

### 3.1. Baseline Characteristics of Testing and Validation Cohorts

**Figure 1:**
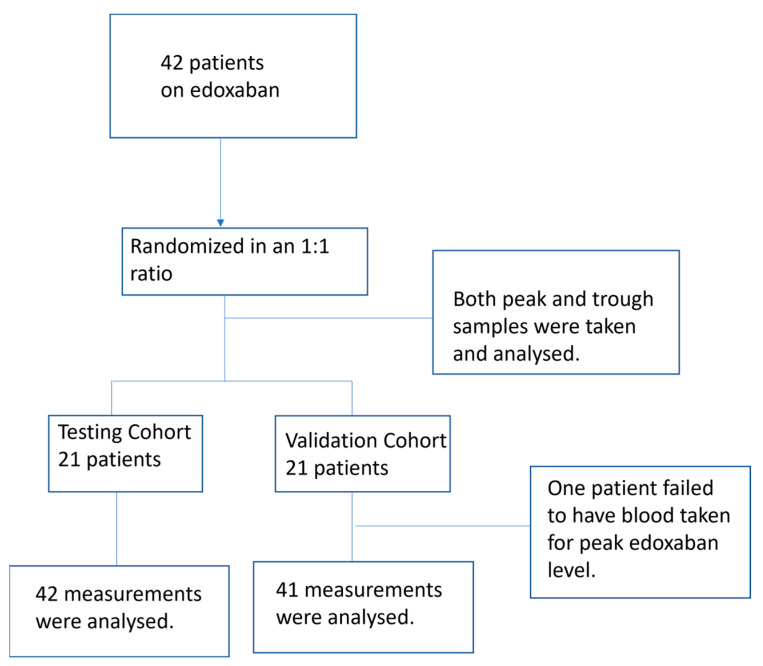
The overall study design of testing and validation cohorts.

A total of 42 patients were recruited during the study period and randomly assigned to testing or validation cohorts in a 1:1 ratio (Figure 1). Baseline characteristics of both cohorts are shown in Table 1. Baseline demographics and medical comorbidities were comparable in both cohorts. The trough plasma edoxaban level and anti-Xa activity of the testing cohort were lower than those of the validation cohort.

### 3.2. Relationship Between Plasma Edoxaban Level and PT, APTT, INR, and Heparin-Calibrated Anti-Xa Acitivity

**Figure 2:**
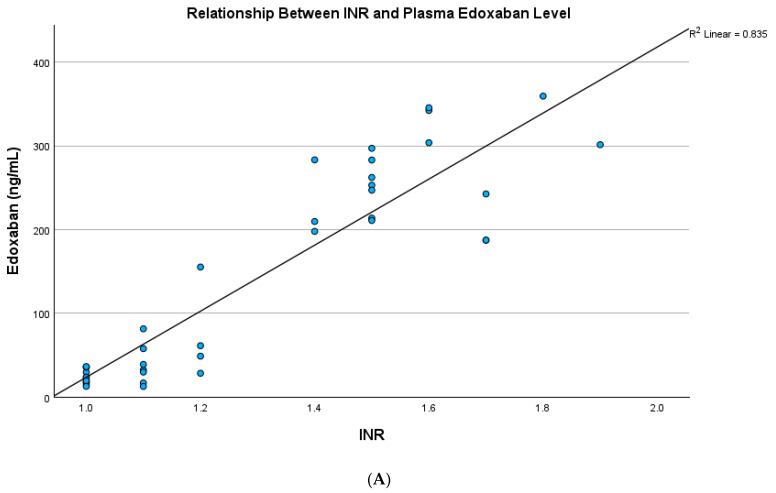

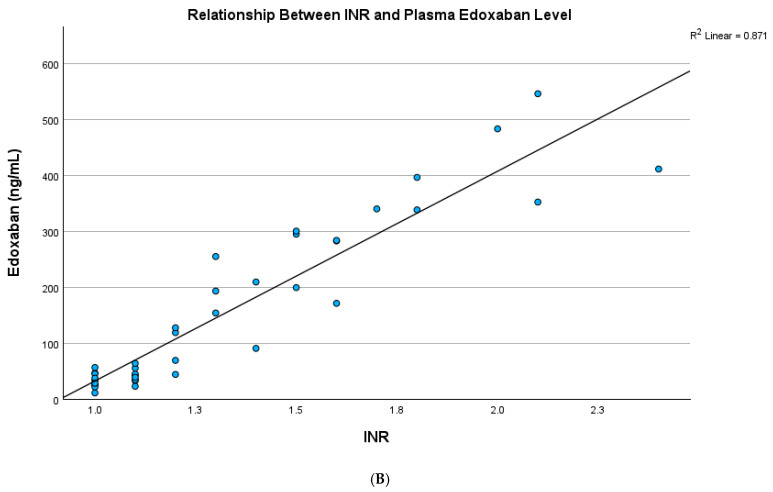
(**A**) The plasma edoxaban level showed a significant and strong relationship with the INR in the testing cohort (R square: 0.835, *p* < 0.001). (**B**) The plasma edoxaban level showed a significant and strong relationship with the INR in the validation cohort (R square: 0.871, *p* < 0.001).

**Figure 3:**
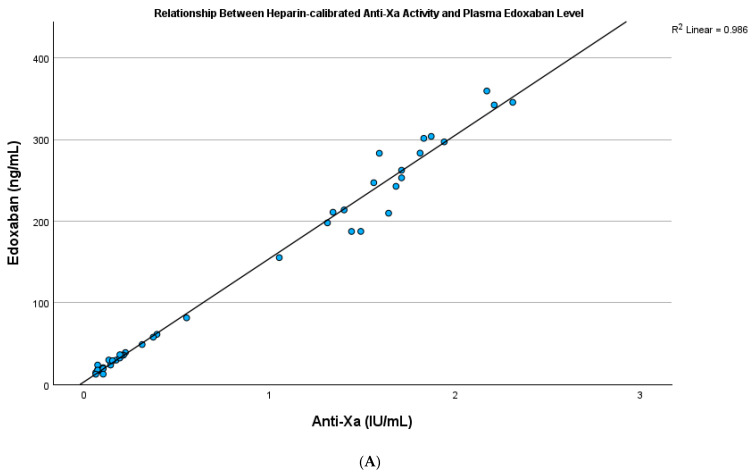

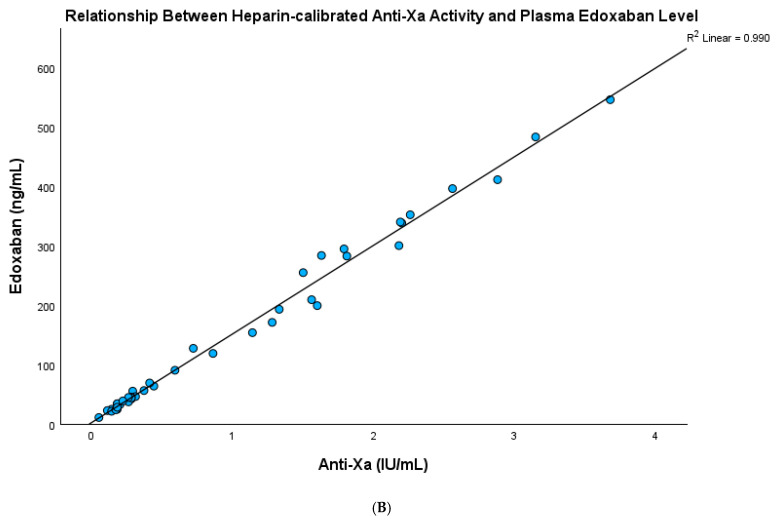
(**A**) The plasma edoxaban level showed a significant and strong relationship with heparin-calibrated anti-Xa activity in the testing cohort (R square: 0.986, *p* < 0.001). (**B**) The plasma edoxaban level showed a significant and strong relationship with heparin-calibrated anti-Xa activity in the validation cohort (R square: 0.990, *p* < 0.001).

The plasma edoxaban level showed a strong positive correlation with PT in both testing and validation cohorts (R square: 0.819, *p* < 0.001; R square: 0.879, *p* < 0.001, respectively) (Supplementary Figure S1A,B) and a strong positive correlation with the INR in both testing and validation cohorts (R square: 0.835, *p* < 0.001; R square: 0.871, *p* < 0.001, respectively) (Figure 2A,B). The plasma edoxaban level showed a significant but rather weak correlation with APTT in both testing and validation cohorts (R square: 0.629, *p* < 0.001; R square: 0.517, *p* < 0.001, respectively) (Supplementary Figure S2A,B) but a strong positive correlation with anti-Xa activity (R square: 0.986, *p* < 0.001; R square: 0.990, *p* < 0.001, respectively) (Figure 3A,B).

### 3.3. Derivation of a Prediction Formula from the INR and Its Validation

Since the plasma edoxaban level showed a strong correlation with the INR, we tried building a formula to predict a plasma edoxaban level based on the INR value by using the curve estimation function of the computer software. The quadratic model had the highest R square value (R square: 0.844, *p* < 0.001) among all models tested and so was used as the basis of the prediction formula:

Plasma edoxaban level = − 784.19 + 1037.55 (INR) − 238.93 (INR)^2^

The root mean square of error (RMSE) of the testing cohort by this formula was 44.91 ng/mL. When this prediction formula was applied to the validation cohort, the RMSE was 59.87 ng/mL. The Bland–Altman plot did not show a statistically significant difference between the measured plasma edoxaban level and that derived from the prediction formula in either cohort. The 95% confidence interval (CI) of the limit of agreement for the testing and validation cohorts were −90.23 to 87.88 ng/mL and −100.02 to 130.01 ng/mL respectively (Supplementary Figure S3A,B).

The plasma DOAC level below 100 ng/mL was considered safe for thrombolytic therapy in patients with ischemic stroke who are taking DOACs according to the results of a study [7]. Using the prediction formula, an INR ≥ 1.2 would represent a plasma edoxaban level of 100 ng/mL and would serve as the cut-off level for administrating intravenous thrombolytic agents to patients presenting with ischemic stroke. The sensitivity and specificity were 100% and 87.0% (95% C.I.: 73.2–100%), respectively, in the testing cohort. In the validation cohort, the sensitivity and specificity were 100% and 86.4% (95% C.I.: 72.0–100%) using the INR-derived prediction formula. (Supplementary Table S1A,B).

When patients present with bleeding complications or urgent need for an invasive procedure, a plasma level < 50 ng/mL would be a cut-off for administration of antidotes or performing such invasive procedures [5]. An INR value ≥ 1.1 would predict a plasma edoxaban level of 50 ng/mL. By using the INR-derived prediction formula, the sensitivity and specificity were 100% and 65% (95% C.I.: 44.1–85.9%), respectively, while the sensitivity and specificity in the validation cohort were 95.8% (95% C.I: 87.8–100%) and 58.8% (95% C.I.: 35.4–82.2%), respectively (Supplementary Table S2A and S2B).

### 3.4. Derivation of Prediction Formula from Heparin-Calibrated Anti-Xa Acitivity and Its Validation

Since there was a strong correlation between the plasma edoxaban level and heparin-calibrated anti-Xa activity, a formula to predict plasma edoxaban level was devised using heparin-calibrated anti-Xa activity. A linear model was chosen due to the highest R square value (R square: 0.986, *p* < 0.001):

Plasma edoxaban level = 3.85 + 150.71 (Anti-Xa activity)

The RMSE was 14.24 ng/mL in the testing cohort. When applied to the validation cohort, the RMSE was 15.04 ng/mL. There was no statistically significant difference between the measured plasma edoxaban level and the value calculated from the prediction formula by the Bland–Altman plot. The 95% confidence interval (CI) of the limit of agreement for the testing and validation cohorts were −28.24 to 28.24 ng/mL and −26.88 to 31.94 ng/mL, respectively (Supplementary Figure S4A,B).

Heparin-calibrated anti-Xa activity ≥ 0.64 predicted a plasma edoxaban level > 100 ng/mL, which precluded safe administration of thrombolytic therapy to patients presenting with ischemic stroke [7]. The prediction formula had 100% sensitivity and specificity in the testing and validation cohorts (Supplementary Table S3A,B).

Regarding treatment decisions for patients presenting with bleeding complications or prior to invasive procedures, anti-Xa activity ≥ 0.31 predicted a plasma edoxaban level > 50 ng/mL. The prediction formula had 100% sensitivity and specificity in the testing cohort but only 95.8% (95% C.I.: 87.8–100%) and 93.8% (95% C.I.: 81.9–100%), respectively, in the validation cohort (Supplementary Table S4A,B).

### 3.5. Utilization of Prediction Formula in Real World Clinical Situation

Our diagnostic laboratory started launching the plasma edoxaban assay in 2020, and the test has been accredited by the College of American Pathologists (CAP). A total of 20 patients with 27 tests were requested by clinicians during the study period. No patients received any blood products prior to blood sampling. Table 2 shows the baseline characteristics of these patients. All patients were commenced on edoxaban with the indication of stroke prophylaxis in AF. Most clinical requests for the measurement of plasma edoxaban level were made prior to an invasive procedure. Only one patient (ER17) with ischemic stroke required urgent intravenous thrombolysis (Supplementary Table S5).

The RMSE was 95.96 ng/mL using the INR-derived prediction formula, which was greater than that of the testing and validation cohorts. The RMSE remained low when applying the heparin-calibrated anti-Xa activity-derived formula (13.70 ng/mL).

Regarding the decision of administering intravenous thrombolysis due to ischemic stroke (100 ng/mL), the INR-derived prediction formula had a sensitivity and specificity of 100% and 38.1% (95% C.I.: 17.3–58.9%), respectively (Supplementary Table S6). Nonetheless, when the INR value was ≥1.5, the specificity reached 100%. The sensitivity and specificity were both 100% using the heparin-calibrated anti-Xa activity-derived formula (Table 3).

Regarding the level that would cause concern about bleeding complications or prior to performing an invasive procedure (50 ng/mL), the INR-derived formula had a sensitivity of 100% but a very low specificity at 18.8% (95% C.I: 0–37.9%) (Supplementary Table S7). The sensitivity and specificity were 90.9% (95% C.I.: 73.9–100%) and 100%, respectively, using the heparin-calibrated anti-Xa activity-derived prediction formula (Table 4).

### 3.6. Proposed Workflow in Real Clinical Practice

**Figure 4:**
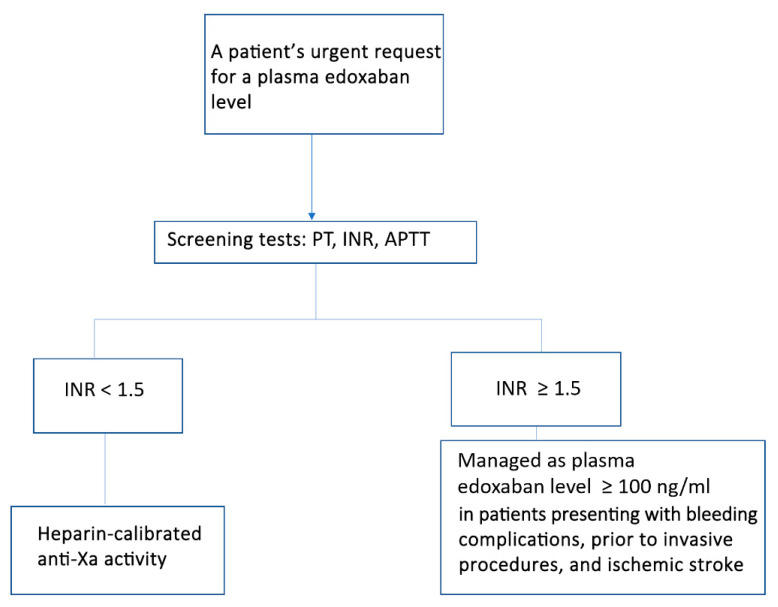
The proposed workflow in our diagnostic laboratory upon receiving urgent requests for plasma edoxaban levels.

The above study demonstrated that although an INR level ≥ 1.2 had 100% sensitivity, the specificity was as low as 38.1% in real patients’ series. An INR ≥ 1.5 increased the specificity to 100% in the testing and validation cohorts, as well as real-life patients’ series. The heparin-calibrated anti-Xa activity-derived formula continued to have high sensitivity and a high specificity with great accuracy in calculating the plasma edoxaban level. Therefore, we can assume that all cases with the INR ≥ 1.5 have a plasma edoxaban level ≥ 100 ng/mL. These patients may require antidote administration or withholding intravenous thrombolytic therapy when they present in clinical emergencies. All patients with the INR value < 1.5 would require heparin-calibrated anti-Xa activity to be measured for the calculation of the plasma edoxaban level (Figure 4).

## 4. Discussion

We successfully built formulae from the INR and heparin-calibrated anti-Xa activity to predict the plasma edoxaban level. The formulae were validated independently in a validation cohort and real-life clinical requests for plasma edoxaban levels. The prediction formulae could be used to estimate plasma edoxaban levels and aid in decision-making in emergency situations, such as bleeding complications, prior to urgent invasive procedures, or administration of intravenous thrombolytics in patients with ischemic stroke. Patients with a plasma DOAC level ≤ 50 ng/mL could safely undergo medium-to-high bleeding risk procedures [21]. Moreover, a plasma DOAC level > 50 ng/mL was proposed as the threshold at which urgent administration of an antidote is required in patients presenting with bleeding complications in certain clinical situations [22]. A plasma DOAC level below 100 ng/mL was proposed as a safe cut-off level at which intravenous thrombolytics would be administered to patients presenting with ischemic stroke [5,7]. Therefore, our study assessed the performance of the prediction formulae at those decision-making plasma DOAC levels. Our pilot study provides a great insight into the use of simple coagulation screening tests and heparin-calibrated anti-Xa measurement in emergency situations when treating patients taking edoxaban.

Routine monitoring of plasma direct oral anticoagulants (DOACs) is not indicated since they are prescribed in a fixed-dose regimen. Nevertheless, the measurement of the plasma DOAC level may be useful in some emergency situations, and the plasma DOAC level should be available within 30 min to facilitate clinical management [6,23]. Moreover, plasma DOAC level measurement is recommended before the administration of antidotes [24]. Although laboratory tests that quantify the plasma DOAC level are valuable to clinical management [25], they are not generally readily available in a 24/7 fashion [26]. The development of a simple prediction formula from the INR or heparin-calibrated anti-Xa assay could address this unmet need. Our pilot study serves as a schema for other units to generate a prediction formula from their own testing platform, and they can then follow the workflow proposed in managing these clinical requests.

The response of routine coagulation screening tests to different plasma edoxaban levels varies and is dependent on the reagents used. Studies have shown a wide variation in the response of the PT ratio to edoxaban; some reagents are quite sensitive to the presence of edoxaban, while others show little change in the PT ratio, even with a high plasma edoxaban concentration [5,27]. Nevertheless, with the use of an appropriate PT reagent, PT remains a good alternative to reflect the plasma edoxaban level [28]. A systematic review also concluded that PT showed a linear concentration-dependent relationship with the plasma edoxaban level, although the response varied widely among different reagents [23]. On the other hand, APTT was much less sensitive to the presence of edoxaban, similar to our findings [29]. It would be optimal to choose a PT reagent that is more sensitive to the presence of edoxaban when applying the prediction formula generated from the INR value.

Although the INR is intended to be used to assess the response to warfarin, its use in predicting the plasma rivaroxaban level has been explored because the INR shows little variation across different PT reagents. [15,30,31]. We found that an INR-derived prediction formula was able to calculate the plasma edoxaban level, albeit with a broad error range. Nevertheless, the formula had a strength in the clinical utility of determining the cut-off plasma edoxaban level to aid in decision-making during medical emergencies. However, the specificity of the INR-derived formula was rather low in real-life situations. The reason was most probably due to prolonged PT in some patient samples for reasons unrelated to edoxaban, e.g., clotting derangement due to disseminated intravascular coagulation. Moreover, the wide error range of the calculated plasma edoxaban level from the formula also contributed to the reduced specificity of the prediction, particularly at a lower range of the plasma edoxaban level.

A chromogenic anti-Xa assay using drug-specific calibrators is a common technique to precisely quantify direct anti-Xa inhibitors. Heparin-calibrated anti-Xa activity utilizes the same principle as a drug-specific chromogenic anti-Xa assay for direct anti-Xa inhibitors. The ENGAGE-AF-TIMI-48 study showed a high concordant rate between heparin-calibrated anti-Xa activity and the drug level measured by the chromogenic anti-Xa assay using a drug-specific calibrator, and the findings were supported by other studies [32,33,34]. The approach of the universal heparin-calibrated anti-Xa assay in calculating the plasma level of all direct anti-Xa inhibitors was successful, as demonstrated in other single-center and multi-center studies [17,35]. We also found an excellent correlation between heparin-calibrated anti-Xa activity and the plasma edoxaban level measured by a drug-specific chromogenic assay in our study with a narrow error range obtained. This transformed into a very high sensitivity and specificity in predicting the decision-making plasma edoxaban level in various clinical emergencies. The technical difficulties in setting up the calibration curve for individual drug-specific chromogenic anti-Xa assays and performing quality control measures hampered a rapid turnaround time for such assays, particularly in those small-scale diagnostic laboratories with limited manpower and technical expertise. Moreover, the small test volume of individual-specific DOAC assays increases the cost of setting up the test [13,14,36]. Furthermore, the lack of an FDA-approved plasma DOAC assay kit imposes barriers to diagnostic laboratories in the US to implement drug-specific anti-Xa assays due to extensive clinical validation requirements for laboratory-developed tests [37]. Therefore, the universal heparin-calibrated anti-Xa assay is an attractive option in the meantime. The assay is convenient to set up even for small diagnostic laboratories. The low specificity of an INR-derived formula to predict the decision-making plasma edoxaban level was the major limitation. The heparin-calibrated anti-Xa activity could complement the INR-derived prediction formula. The novel approach of our proposed workflow takes advantage of both the INR-derived formula (simple, rapid, and easily available) and the heparin-calibrated anti-Xa activity-derived formula (a high degree of accuracy). The need to perform more sophisticated heparin-calibrated anti-Xa assays for the calculation of the plasma edoxaban level in clinical emergencies would be much reduced if the INR-derived formula were used in all patients with an INR ≥ 1.5 as the cut-off value. This would save manpower costs for running the calibrators, quality control materials, setting up the calibration curve, and running the test of heparin-calibrated anti-Xa assays and would reserve the manpower for performing other emergency laboratory services.

Although our pilot study establishes a novel approach to a workflow in handling urgent requests for the plasma edoxaban level, this study had several limitations. First, the sample size of the study was rather small, and the findings may not be representative. However, our findings could set the stage for future clinical studies. Second, those patients included in the study of real-life situations did not have a wide range of medical conditions, especially those that would have given rise to a deranged clotting profile such as disseminated intravascular coagulation. In addition, the INR-derived formula is not applicable to patients with liver disease, which could be increasingly problematic due to the emergence of the evidence of prescribing edoxaban to patients with liver cirrhosis [38,39]. Also, the sensitivity of the INR-derived prediction formula may be much reduced if patients have received prior therapy to correct abnormal clotting, e.g., plasma transfusion. It is vital to determine the transfusion history and history of prior therapy for deranged clotting before applying the INR-derived prediction formula. Third, the INR-derived formula had a wide error range, and the accuracy may not be adequate for the exact quantification of drug level. Furthermore, this study was conducted on one type of PT reagent only, which was sensitive to edoxaban. The results of this study may not be able to generalize to all PT reagents available on the market. Also, this study only evaluated edoxaban but not all other direct Xa inhibitors. Moreover, the proposed workflow is not applicable to patients taking dabigatran since the heparin-calibrated anti-Xa assay is not valid in this situation. Furthermore, one of the drawbacks of the proposed workflow is that the diagnostic laboratories need to spend time and effort to recruit adequate number of patients in order to set up prediction formulae, though the plasma samples could be archived for resetting the formulae in the future due to various circumstances, such as changing the reagent lot. Lastly, it is a single-center study, and the results may not be generalized to all centers.

In summary, despite the above limitations, our pilot study demonstrated the successful generation of a prediction formula for the plasma edoxaban level based on the INR and heparin-calibrated anti-Xa activity. Our proposed novel two-tier workflow can help streamline the process to estimate a plasma edoxaban level with a consequent rapid turnaround time and high sensitivity and specificity. Further large-scale multi-center studies that utilize multiple PT reagents are essential to evaluate the clinical utility of this approach. Also, the study patients should include patients taking other DOACs in addition to edoxaban. Moreover, patients with a wide range of medical history need to be recruited in future studies to consolidate the applicability of the formulae in a wide variety of real-world clinical situations.

## Figures and Tables

**Table 1:** Baseline characteristics of study patients in the testing and validation cohorts.

	Testing Cohort (*n* = 21)	Validation Cohort (*n* = 21)	*p*-Value	Overall (*n* = 42)
Demographic information				
Sex (male) (%)	14 (66.7%)	9 (42.9%)	0.121	23 (54.8)
Mean age (years) (range)	73.1 (56–85)	72.5 (49–88)	0.834	72.8 (49–88)
Body weight, (kg), mean (range)	62.0 (39.8–86.5)	62.0 (38.6–85.6)	0.988	62.0 (38.6–86.5)
Dosage	66.7% (30 mg) 33.3% (60 mg)	38.1% (30 mg) 61.9% (60 mg)	0.064	52.4% (30 mg) 47.6% (60 mg)
CHADS2, median (IQR)	2.0 (2.0)	2.0 (2.0)	0.651	2.0 (2.0)
Laboratory parameters				
eGFR by MDRD equation, median (IQR)	68.00 (56–79)	61.00 (46–79)	0.345	66.50 (48–78)
Creatinine (umol/L), median (IQR)	92.19 (76–105)	94.00 (67–132)	0.615	94.00 (71–117)
CrCl (mL/min), median (IQR)	54.81 (40–64)	46.95 (39–66)	0.606	52.11 (39–65)
Peak PT (s), median (IQR)	16.5 (15.5–18.3)	17.4 (14.6–19.9)	0.426	16.7 (15.1–18.6)
Trough PT (s), median (IQR)	11.8 (11.4–12.5)	11.8 (11.4–12.4)	0.870	11.8 (11.4–12.4)
Overall PT (s), median (IQR)	13.6 (11.8–16.5)	13.1 (11.8–17.4)	0.816	13.3 (11.8–16.7)
Peak APTT (s), median (IQR)	35.5 (33.2–37.5)	36.0 (33.5–43.6)	0.389	35.5 (33.3–38.7)
Trough APTT (s), median (IQR)	28.5 (27.2–30.0)	29.7 (26.9–31.9)	0.232	29.0 (27.1–30.7)
Overall APTT (s), median (IQR)	31.2 (28.4–35.5)	32.5 (29.2–36.6)	0.325	31.9 (28.5–35.9)
Peak INR, mean (range)	1.5 (1.4–1.7)	1.6 (1.3–1.8)	0.562	1.5 (1.4–1.7)
Trough INR, median (IQR)	1.0 (1.0–1.1)	1.0 (1.0–1.1)	0.610	1.0 (1.0–1.1)
Overall INR, median (IQR)	1.2 (1.0–1.5)	1.2 (1.0–1.6))	0.726	1.2 (1.0–1.5)
Peak anti-Xa activity (IU/mL), median (IQR)	1.64 (1.37–1.85)	1.71 (1.29–2.25)	0.434	1.64 (1.34–2.18)
Trough anti-Xa activity (IU/mL), median (IQR)	0.14 (0.08–0.20)	0.26 (0.18–0.31)	0.001	0.18 (0.12–0.28)
Overall anti-Xa activity (IU/mL), median (IQR)	0.38 (0.14–1.65)	0.52 (0.23–1.75)	0.149	0.43 (0.18–1.65)
Peak edoxaban (ng/mL), median (IQR)	247.1 (192.7–299.2)	283.6 (176.9–349.6)	0.481	255.2 (190.4–321.2)
Trough edoxaban (ng/mL), median (IQR)	28.4 (17.6–35.8)	37.6 (25.1–51.0)	0.014	31.1 (22.6–44.4)
Medical comorbidity (%)				
Diabetes mellitus (%)	5 (23.8%)	7 (33.3%)	0.495	12 (28.6%)
Heart failure (%)	3 (14.3%)	3 (14.3%)	1.000	6 (14.3%)
Hyperlipidemia (%)	9 (42.9%)	10 (47.6%)	0.757	19 (45.2%)
Hypertension (%)	13 (61.9%)	15 (71.4%)	0.513	28 (66.7%)
IHD (%)	6 (28.6%)	8 (38.1%)	0.513	14 (33.3%)
History of CVA or TIA (%)	4 (19.0%)	4 (19.0%)	1.000	8 (19.0%)

**Table 2:** Baseline characteristics of real-life patients of clinical requests for the plasma edoxaban level. (Total of 20 patients with 27 clinical requests).

Demographic Information	
Sex (male) (%)	11 (64.7%)
Age, years old, mean (range)	76.6 (33–92)
Dosage	29.4% (15 mg) 35.3% (30 mg) 35.3% (60 mg)
Laboratory parameters	
PT (s), median (IQR)	14.5 (12.8–16.6)
APTT (s), median (IQR)	1.3 (1.2–1.5)
INR, median (IQR)	31.2 (29.4–32.9)
Anti-Xa activity (IU/mL), median (IQR)	0.25 (0.09–0.65)
Plasma edoxaban (ng/mL), median (IQR)	44.9 (20.7–99.7)

**Table 3:** The diagnostic performance of using a heparin-calibrated anti-Xa activity-derived formula to determine the cut-off plasma edoxaban level for administrating intravenous thrombolysis in patients presenting with ischemic stroke (100 ng/mL). The data were generated from real-life clinical requests of the plasma edoxaban level. From the prediction formula, the anti-Xa activity of ≥0.64 would predict the plasma edoxaban level of 100 ng/mL. Both the sensitivity and specificity were 100%.

	Plasma Edoxaban Level > 100 ng/mL	Plasma Edoxaban Level ≤ 100 ng/mL	Total
Anti-Xa ≥ 0.64	6	0	6
Anti-Xa < 0.64	0	21	21
Total	6	21	27

**Table 4:** The diagnostic performance of using heparin-calibrated anti-Xa activity-derived formula to determine the cut-off plasma edoxaban level for treating patients with bleeding complication or prior invasive procedures (50 ng/mL). The data were generated from real-life clinical requests from patients. From the prediction formula, the anti-Xa activity of ≥0.31 would predict the plasma edoxaban level of 50 ng/mL. The sensitivity and specificity were 90.9% (95% C.I.: 73.9–100%) and 100%, respectively.

	Plasma Edoxaban Level > 50 ng/mL	Plasma Edoxaban Level ≤ 50 ng/mL	Total
Anti-Xa ≥ 0.31	10	0	10
Anti-Xa < 0.31	1	16	17
Total	11	16	27

## Data Availability

The data are not publicly available due to privacy concerns. The data presented in this study are available on request from the corresponding author.

## Supplementary Materials

The following supporting information can be downloaded at: https://www.mdpi.com/article/10.3390/jcm14031006/s1, Figure S1A: The plasma edoxaban level and PT showed strong and significant correlation in testing cohort (R square: 0.819, *p* < 0.001). Figure S1B: The plasma edoxaban level and PT showed strong and significant correlation in validation cohort (R square: 0.879, *p* < 0.001). Figure S2A: The plasma edoxaban level showed a significant relationship with APTT in testing cohort (R square: 0.629, *p* < 0.001). Figure S2B: The plasma edoxaban level showed a significant relationship with APTT in validation cohort (R square: 0.517, *p* < 0.001). Figure S3A: The difference between predicted and measured plasma edoxaban level versus mean plasma edoxaban level in the testing cohort using the INR-derived formula are shown by Bland-Altman plot. The 95% confidence interval (CI) of limit of agreement for testing by INR-derived prediction formula was −90.23 to 87.88 ng/mL. Figure S3B: The difference between predicted and measured plasma edoxaban level versus mean plasma edoxaban level in validation cohort from INR-derived formula are shown by Bland-Altman plot. The 95% confidence interval (CI) of limit of agreement was −100.02 to 130.01 ng/mL. Figure S4A: The difference between predicted and measured plasma edoxaban level versus mean plasma edoxaban level in the testing cohort from heparin calibrated anti-Xa derived formula are shown by Bland-Altman plot. The 95% confidence interval (CI) of limit of agreement was −28.24 to 28.24 ng/mL. Figure S4B: The difference between predicted and measured plasma edoxaban level versus mean plasma edoxaban level in the validation cohort from heparin-calibrated anti-Xa derived formula are shown by Bland-Altman plot. The 95% confidence interval (CI) of limit of agreement was −26.88 to 31.94 ng/mL. Table S1: The diagnostic performance of using INR-derived formula to determine the cut-off plasma edoxaban level for intravenous thrombolytic therapy for patients present with ischaemic stroke (100 ng/mL). From the prediction formula, the INR of ≥1.2 would predict the plasma edoxaban level of 100 ng/mL. The results from (A) Testing cohort showed sensitivity and specificity of 100% and 87.0% (95% C.I.: 73.2–100%) respectively and (B) Validation cohort showed sensitivity and specificity of 100% and 86.4% (95% C.I.: 72.0–100%) respectively. Table S2: The diagnostic performance of using INR-derived formula to determine the cut-off plasma edoxaban level for managing patients with bleeding complications or prior to urgent invasive procedures (50 ng/mL). From the prediction formula, the INR of ≥1.1 would predict the plasma edoxaban level of 50 ng/mL. The results from (A) Testing cohort showed sensitivity and specificity of 100% and 65% (95% C.I.: 44.1–85.9%) respectively and (B) Validation cohort showed sensitivity and specificity of 95.8% (95% C.I: 87.8–100%) and 58.8% (95% C.I.: 35.4–82.2%) respectively. Table S3: The diagnostic performance of using heparin calibrated anti-Xa activity derived formula to determine the cut-off plasma edoxaban level for administrating intravenous thrombolysis in patients presenting with ischaemic stroke (100 ng/mL). From the prediction formula, the anti-Xa activity of ≥0.64 would predict the plasma edoxaban level of 100 ng/mL. The results from (A) Testing cohort showed 100% sensitivity and specificity and (B) Validation cohort also showed 100% sensitivity and specificity. Table S4: The diagnostic performance of using heparin calibrated anti-Xa activity derived formula to determine the cut-off plasma edoxaban level for managing patients with bleeding complication or prior invasive procedures (50 ng/mL). From the prediction formula, the anti-Xa activity of ≥0.31 would predict the plasma edoxaban level of 50 ng/mL. The results from A) Testing cohort showed 100% sensitivity and specificity and B) Validation cohort showed sensitivity and specificity of 95.8% (95% C.I.: 87.8–100%) and 93.8% (95% C.I.: 81.9–100%) respectively. Table S5: Details of clinical indications for requesting plasma edoxaban level. Table S6: The diagnostic performance of using INR-derived formula to determine the cut-off plasma edoxaban level for administrating intravenous thrombolysis in patients presenting with ischaemic stroke (100 ng/mL). The data was generated from real-life clinical requests of plasma edoxaban level. From the prediction formula, the INR value of ≥1.2 would predict the plasma edoxaban level of 100 ng/mL. The sensitivity and specificity were 100% and 38.1% (95% C.I.: 17.3–58.9%) respectively. Table S7: The diagnostic performance of using INR-derived formula to determine the cut-off plasma edoxaban level for managing patients with bleeding complications or prior to urgent invasive procedures (50 ng/mL). The data was generated from real-life clinical requests of patients. From the prediction formula, the INR of ≥1.1 would predict the plasma edoxaban level of 50 ng/mL. The sensitivity was 100%. However, the specificity was rather low (18.8%, 95% C.I: 0–37.9%).

## Abbreviations

Atrial fibrillation, AF; direct oral anticoagulants, DOACs; PT, prothrombin time; APTT, activated partial thromboplastin time; INR, international normalized ratio; ISI, international sensitivity index; RMSE, root mean square error; WHO, World Health Organization; EQAS, external quality assurance scheme; creatinine clearance, CrCl; diabetes mellitus, DM; cerebrovascular accident, CVA; ICSH, International Council for Standardization in Haematology.

## References

[1] Joglar J.A., Chung M.K., Armbruster A.L., Benjamin E.J., Chyou J.Y., Cronin E.M., Lee A.D.L., Goldberger E.Z.D., Gopinathannair R., Writing Committee Members (2024). 2023 ACC/AHA/ACCP/HRS Guideline for the Diagnosis and Management of Atrial Fibrillation: A Report of the American College of Cardiology/American Heart Association Joint Committee on Clinical Practice Guidelines. J. Am. Coll. Cardiol..

[2] Lip G.Y., Agnelli G. (2014). Edoxaban: A focused review of its clinical pharmacology. Eur. Heart J..

[3] Steffel J., Collins R., Antz M., Cornu P., Desteghe L., Haeusler K.G., Oldgren J., Reinecke H., Roldan-Schilling V., Rowell N. (2021). 2021 European Heart Rhythm Association Practical Guide on the Use of Non-Vitamin K Antagonist Oral Anticoagulants in Patients with Atrial Fibrillation. Europace.

[4] Hindley B., Lip G.Y.H., McCloskey A.P., Penson P.E. (2023). Pharmacokinetics and pharmacodynamics of direct oral anticoagulants. Expert Opin. Drug Metab. Toxicol..

[5] Douxfils J., Ageno W., Samama C.M., Lessire S., Ten Cate H., Verhamme P., Dogné J., Mullier F. (2018). Laboratory testing in patients treated with direct oral anticoagulants: A practical guide for clinicians. J. Thromb. Haemost..

[6] Douxfils J., Adcock D.M., Bates S.M., Favaloro E.J., Gouin-Thibault I., Guillermo C., Kawai Y., Lindhoff-Last E., Kitchen S., Gosselin R.C. (2021). 2021 Update of the International Council for Standardization in Haematology Recommendations for Laboratory Measurement of Direct Oral Anticoagulants. Thromb. Haemost..

[7] Seiffge D.J., Traenka C., Polymeris A.A., Thilemann S., Wagner B., Hert L., Müller M.D., Gensicke H., Peters N., Nickel C.H. (2017). Intravenous Thrombolysis in Patients with Stroke Taking Rivaroxaban Using Drug Specific Plasma Levels: Experience with a Standard Operation Procedure in Clinical Practice. J. Stroke.

[8] Berge E., Whiteley W., Audebert H., De Marchis G.M., Fonseca A.C., Padiglioni C., de la Ossa N.P., Strbian D., Tsivgoulis G., Turc G. (2021). European Stroke Organisation (ESO) guidelines on intravenous thrombolysis for acute ischaemic stroke. Eur. Stroke J..

[9] Sin C.F., Wong K.P., Wong H.M., Siu C.W., Yap D.Y.H. (2022). Plasma Rivaroxaban Level in Patients with Early Stages of Chronic Kidney Disease-Relationships with Renal Function and Clinical Events. Front. Pharmacol..

[10] Sin C.F., Wong K.P., Wong T.F., Siu C.W., Yap D.Y.H. (2022). Plasma apixaban levels in Chinese patients with chronic kidney disease-Relationship with renal function and bleeding complications. Front. Pharmacol..

[11] Parasrampuria D.A., Truitt K.E. (2016). Pharmacokinetics and Pharmacodynamics of Edoxaban, a Non-Vitamin K Antagonist Oral Anticoagulant that Inhibits Clotting Factor Xa. Clin. Pharmacokinet..

[12] He L., Kochan J., Lin M., Vandell A., Brown K., Depasse F. (2017). Determination of edoxaban equivalent concentrations in human plasma by an automated anti-factor Xa chromogenic assay. Thromb. Res..

[13] Boissier E., Senage T., Babuty A., Gouin-Thibault I., Rozec B., Roussel J.-C., Sigaud M., Ternisien C., Trossaert M., Fouassier M. (2021). Heparin Anti-Xa Activity, a Readily Available Unique Test to Quantify Apixaban, Rivaroxaban, Fondaparinux, and Danaparoid Levels. Anesth. Analg..

[14] Sarode R. (2019). Direct oral anticoagulant monitoring: What laboratory tests are available to guide us?. Hematol. Am. Soc. Hematol. Educ. Program.

[15] Sin C.F., Wong K.P., Siu C.W., Wong T.F., Wong H.M. (2024). Utilization of international normalized ratio-derived formula to predict plasma rivaroxaban level—Validation study and real-world experience. Int. J. Lab. Hematol..

[16] Hillarp A., Strandberg K., Baghaei F., Fagerberg Blixter I., Gustafsson K.M., Lindahl T.L. (2018). Effects of the oral, direct factor Xa inhibitor edoxaban on routine coagulation assays, lupus anticoagulant and anti-Xa assays. Scand. J. Clin. Lab. Investig..

[17] Willekens G., Studt J., Mendez A., Alberio L., Fontana P., Wuillemin W.A., Schmidt A., Graf L., Gerber B., Bovet C. (2021). A universal anti-Xa assay for rivaroxaban, apixaban, and edoxaban measurements: Method validation, diagnostic accuracy and external validation. Br. J. Haematol..

[18] Michels W.M., Grootendorst D.C., Verduijn M., Elliott E.G., Dekker F.W., Krediet R.T. (2010). Performance of the Cockcroft-Gault, MDRD, and new CKD-EPI formulas in relation to GFR, age, and body size. Clin. J. Am. Soc. Nephrol..

[19] Gosselin R.C., Adcock D.M., Bates S.M., Douxfils J., Favaloro E.J., Gouin-Thibault I., Guillermo C., Kawai Y., Lindhoff-Last E., Kitchen S. (2018). International Council for Standardization in Haematology (ICSH) Recommendations for Laboratory Measurement of Direct Oral Anticoagulants. Thromb. Haemost..

[20] Poller L., Ibrahim S., Keown M., Pattison A., Jespersen J., European Action on Anticoagulation (2011). The prothrombin time/international normalized ratio (PT/INR) Line: Derivation of local INR with commercial thromboplastins and coagulometers—Two independent studies. J. Thromb. Haemost..

[21] Douketis J.D., Spyropoulos A.C., Duncan J., Carrier M., Le Gal G., Tafur A.J., Vanassche T., Verhamme P., Shivakumar S., Gross P.L. (2019). Perioperative Management of Patients with Atrial Fibrillation Receiving a Direct Oral Anticoagulant. JAMA Intern. Med..

[22] Levy J.H., Ageno W., Chan N.C., Crowther M., Verhamme P., Weitz J.I., for the Subcommittee on Control of Anticoagulation (2016). When and how to use antidotes for the reversal of direct oral anticoagulants: Guidance from the SSC of the ISTH. J. Thromb. Haemost..

[23] Samuelson B.T., Cuker A., Siegal D.M., Crowther M., Garcia D.A. (2017). Laboratory Assessment of the Anticoagulant Activity of Direct Oral Anticoagulants: A Systematic Review. Chest.

[24] Gendron N., Billoir P., Siguret V., Le Cam-Duchez V., Proulle V., Macchi L., Boissier E., Mouton C., De Maistre E., Gouin-Thibault I. (2024). Is there a role for the laboratory monitoring in the management of specific antidotes of direct oral anticoagulants?. Thromb. Res..

[25] Connors J.M. (2018). Testing and monitoring direct oral anticoagulants. Blood.

[26] Beyer-Westendorf J., Kohler C. (2023). Direct Oral Anticoagulants: Laboratory Challenges and Antidotes. Hamostaseologie.

[27] Testa S., Dellanoce C., Paoletti O., Cancellieri E., Morandini R., Tala M., Zambelli S., Legnani C. (2019). Edoxaban plasma levels in patients with non-valvular atrial fibrillation: Inter and intra-individual variability, correlation with coagulation screening test and renal function. Thromb. Res..

[28] Patel J.P., Chitongo P.B., Dighe P., Roberts L.N., Vadher B., Patel R.K., Arya R. (2019). Prothrombin times in the presence of edoxaban—In-vivo experience from King’s College hospital. Br. J. Haematol..

[29] Morishima Y., Kamisato C. (2015). Laboratory measurements of the oral direct factor Xa inhibitor edoxaban: Comparison of prothrombin time, activated partial thromboplastin time, and thrombin generation assay. Am. J. Clin. Pathol..

[30] Van den Besselaar A.M., Barrowcliffe T.W., Houbouyan-Reveillard L.L., Jespersen J., Johnston M., Poller L., Tripodi A., On behalf of the Subcommittee on Control of Anticoagulation of the Scientific and Standardization Committee of the ISTH (2004). Guidelines on preparation, certification, and use of certified plasmas for ISI calibration and INR determination. J. Thromb. Haemost..

[31] Ofek F., Barchel D., Perets N., Ziv-Baran T., Mahajna A., Filipovich-Rimon T., Garach-Jehoshua O., Berlin M., Berkovitch M. (2019). International Normalized Ratio as a Screening Test for Assessment of Anticoagulant Activity for Patients Treated with Rivaroxaban or Apixaban: A Pilot Study. Front. Pharmacol..

[32] Ruff C.T., Giugliano R.P., Braunwald E., Morrow D.A., Murphy S.A., Kuder J.F., Deenadayalu N., Jarolim P., Betcher J., Shi M. (2015). Association between edoxaban dose, concentration, anti-Factor Xa activity, and outcomes: An analysis of data from the randomised, double-blind ENGAGE AF-TIMI 48 trial. Lancet.

[33] Singh J., Ong D.M., Wallis A., Kelsey G., Tran H. (2019). Anti-Xa levels with low molecular weight heparin calibrator can be used to exclude significant apixaban effect. Pathology.

[34] Lim M.S., Hayes R., Sharma A., Kitiponchai T., Mohamed M., McRae S. (2022). Prospective cohort study on the use of low molecular weight heparin calibrated anti-Xa assay for measurement of direct oral Xa inhibitors in ex vivo patient samples. Pathology.

[35] Meihandoest T., Studt J.D., Mendez A., Alberio L., Fontana P., Wuillemin W.A., Schmidt A., Graf L., Gerber B., Amstutz U. (2022). Accuracy of a Single, Heparin-Calibrated Anti-Xa Assay for the Measurement of Rivaroxaban, Apixaban, and Edoxaban Drug Concentrations: A Prospective Cross-Sectional Study. Front. Cardiovasc. Med..

[36] Wright C., Brown R., Cuker A. (2017). Laboratory measurement of the direct oral anticoagulants: Indications and impact on management in clinical practice. Int. J. Lab. Hematol..

[37] Qiao J., Tran M.H. (2024). Challenges to Laboratory Monitoring of Direct Oral Anticoagulants. Clin. Appl. Thromb. Hemost..

[38] Tadokoro T., Tani J., Manabe T., Takuma K., Nakahara M., Oura K., Mimura S., Fujita K., Nomura T., Morishita A. (2024). Effectiveness of edoxaban in portal vein thrombosis associated with liver cirrhosis. Sci. Rep..

[39] Portela C.P., Gautier L.A., Zermatten M.G., Fraga M., Moradpour D., Calderara D.B., Aliotta A., Veuthey L., De Gottardi A., Stirnimann G. (2024). Direct oral anticoagulants in cirrhosis: Rationale and current evidence. JHEP Rep..

