# Supplementary material for: A Pilot Study to Evaluate an International Normalized Ratio-Derived Formula in Combination with Heparin-Calibrated Anti-Xa Activity in Calculating a Plasma Edoxaban Level
Source: J Clin Med. 2025 Feb 5;14(3):1006. doi: 10.3390/jcm14031006 (PMC11818660; doi:10.3390/jcm14031006)
Supplement: Supplementary file 1 [file jcm-14-01006-s001.zip › jcm-3167873-supplementary.pdf]

Supplementary Figure S1A: The plasma edoxaban level and PT showed strong and significant correlation in testing cohort (R square: 0.819, p < 0.001).

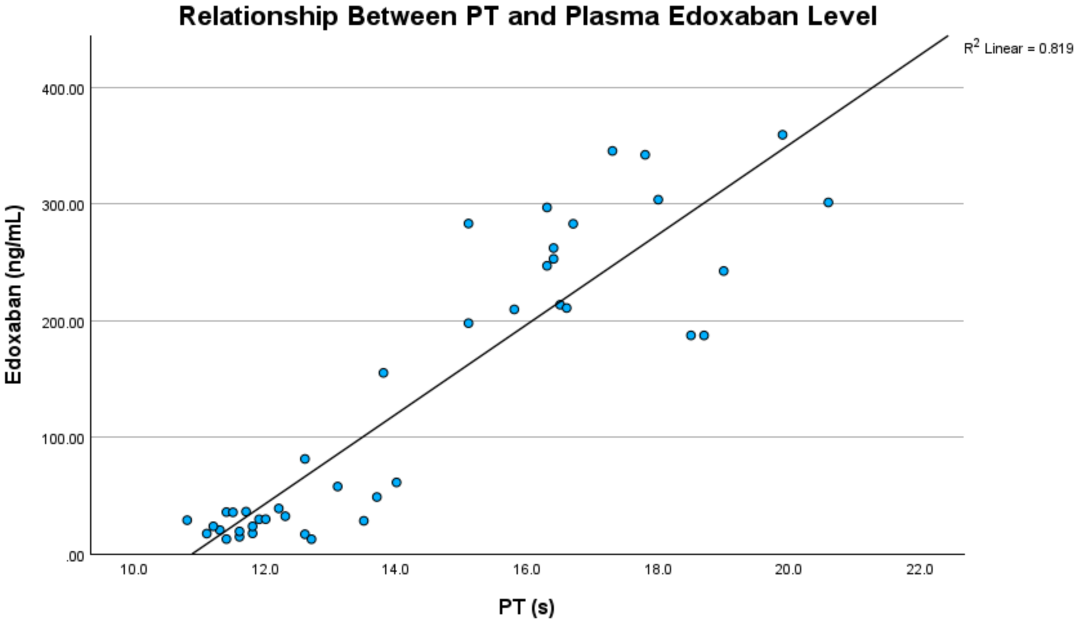

Supplementary Figure S1B: The plasma edoxaban level and PT showed strong and significant correlation in validation cohort (R square: 0.879, p < 0.001).

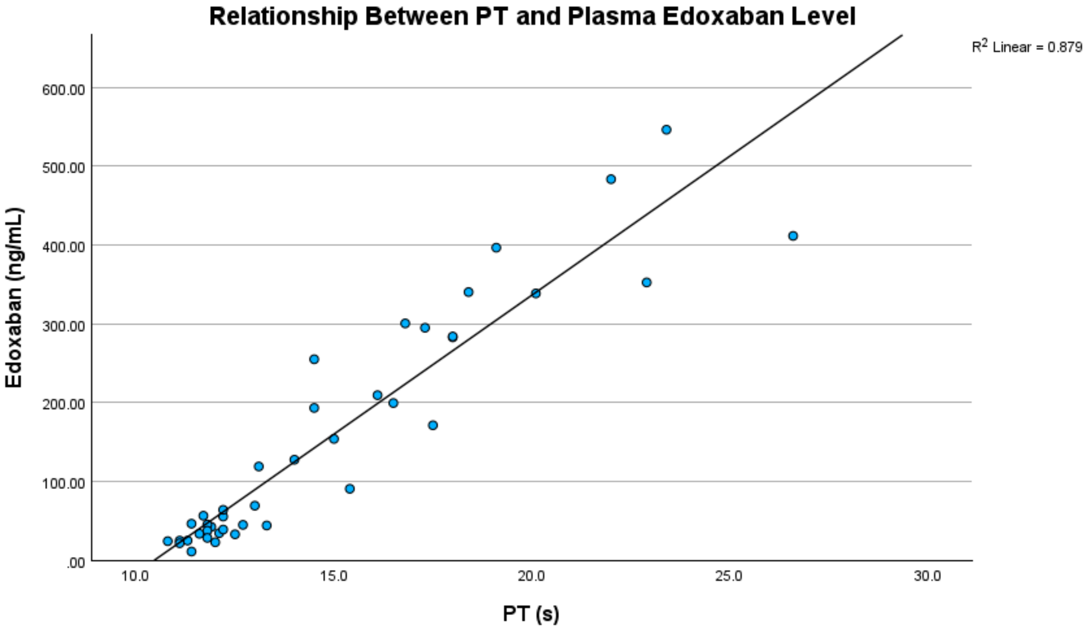

Supplementary Figure S2A: The plasma edoxaban level showed a significant relationship with APTT in testing cohort (R square: 0.629, p < 0.001).

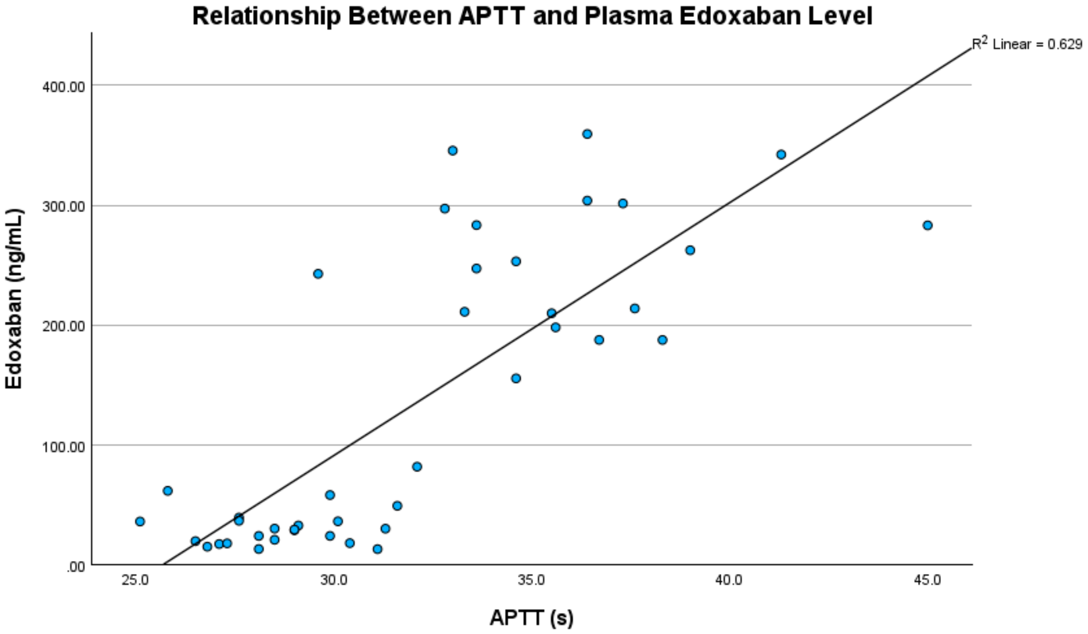

Supplementary Figure S2B: The plasma edoxaban level showed a significant relationship with APTT in validation cohort (R square: 0.517, p < 0.001).

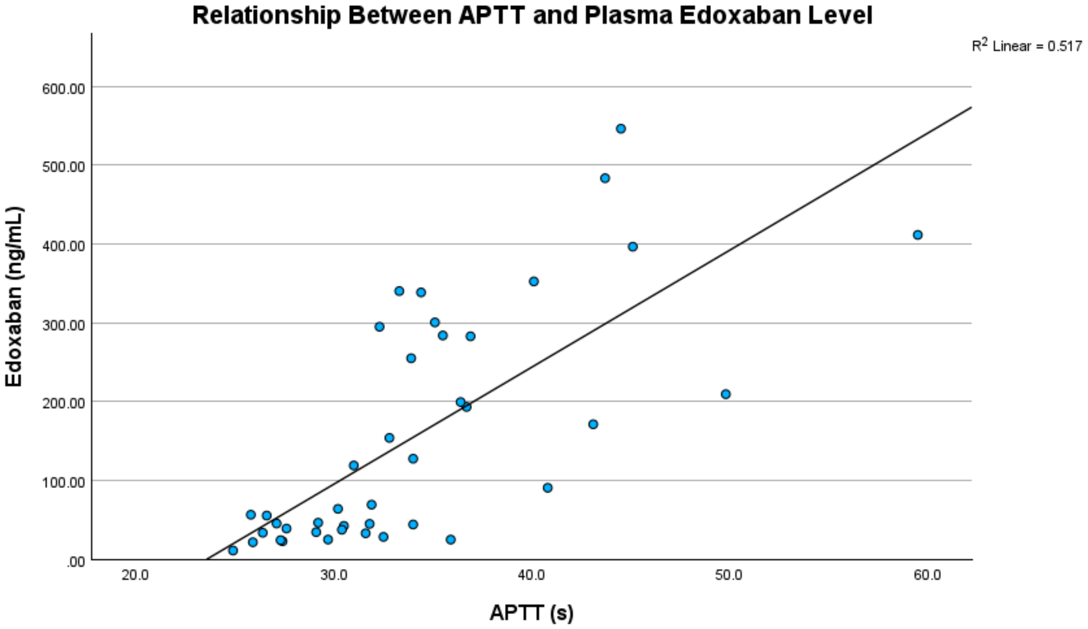

Supplementary Figure S3A: The difference between predicted and measured plasma edoxaban level versus mean plasma edoxaban level in the testing cohort using the INR-derived formula are shown by Bland-Altman plot. The 95% confidence interval (CI) of limit of agreement for testing by INR-derived prediction formula was -90.23 to 87.88 ng/ml.

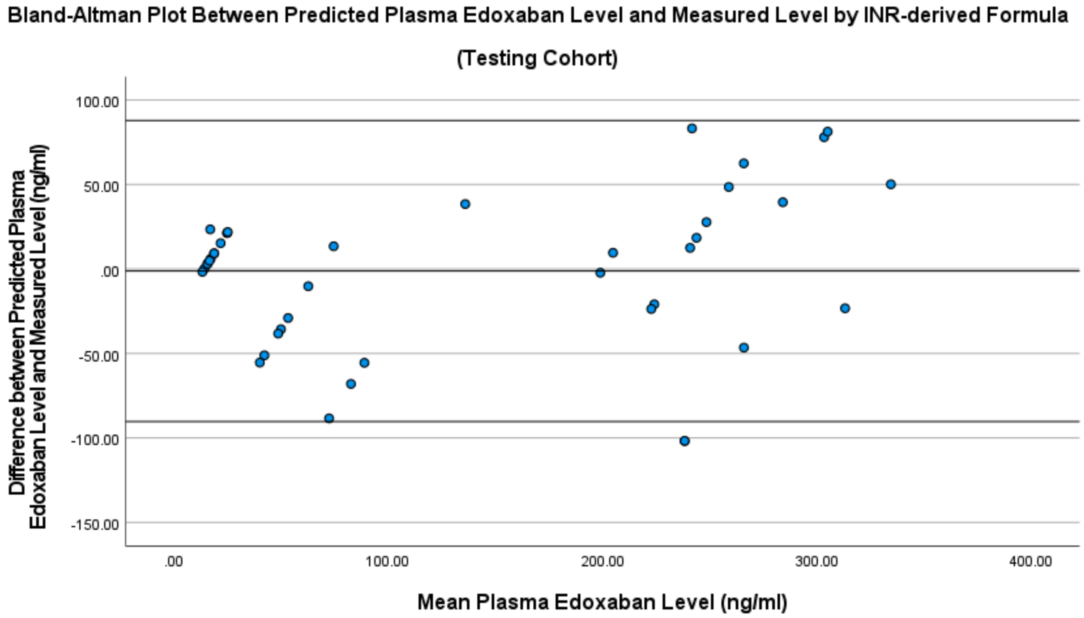

Supplementary Figure S3B: The difference between predicted and measured plasma edoxaban level versus mean plasma edoxaban level in validation cohort from INR-derived formula are shown by Bland-Altman plot. The 95% confidence interval (CI) of limit of agreement was -100.02 to 130.01 ng/ml.

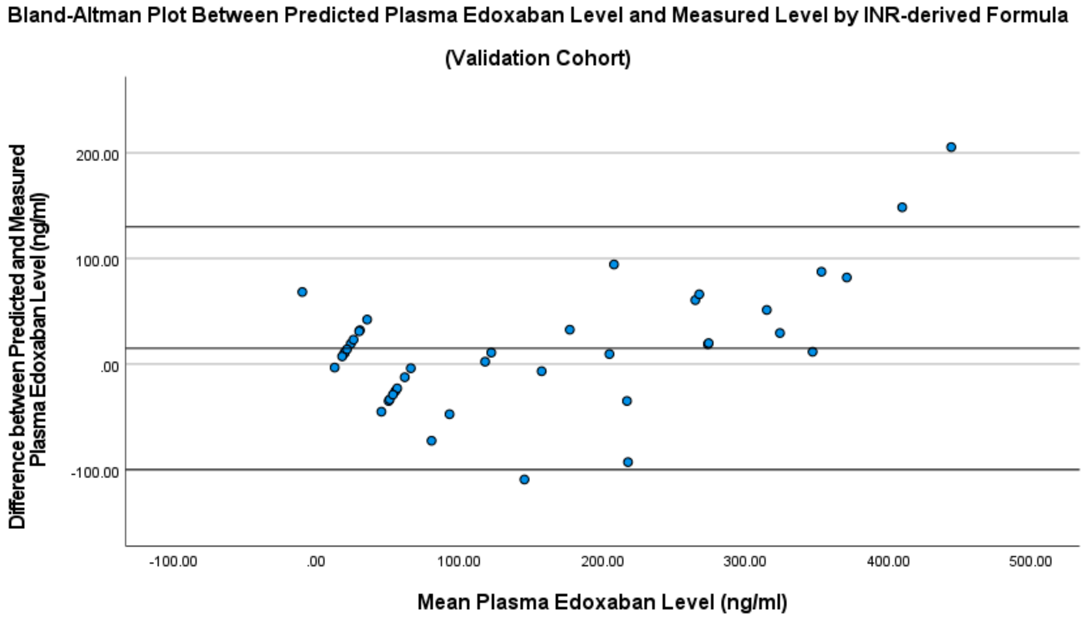

Supplementary Figure S4A: The difference between predicted and measured plasma edoxaban level versus mean plasma edoxaban level in the testing cohort from heparin calibrated anti-Xa derived formula are shown by Bland-Altman plot. The 95% confidence interval (CI) of limit of agreement was -28.24 to 28.24 ng/ml.

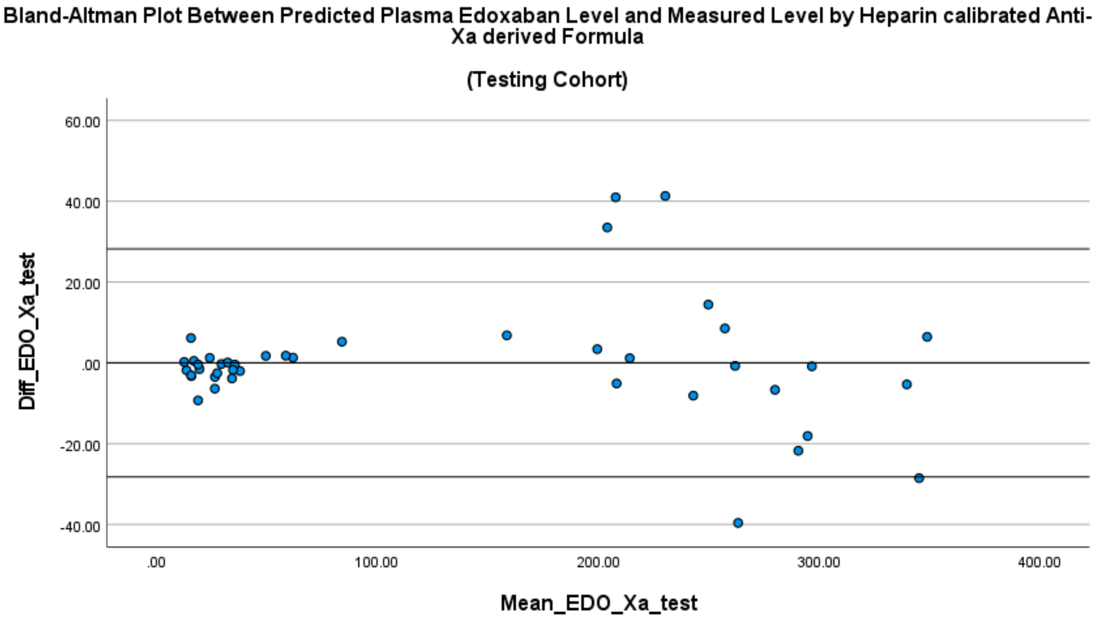

Supplementary Figure S4B: The difference between predicted and measured plasma edoxaban level versus mean plasma edoxaban level in the validation cohort from heparin-calibrated anti-Xa derived formula are shown by Bland-Altman plot. The 95% confidence interval (CI) of limit of agreement was -26.88 to 31.94 ng/ml.

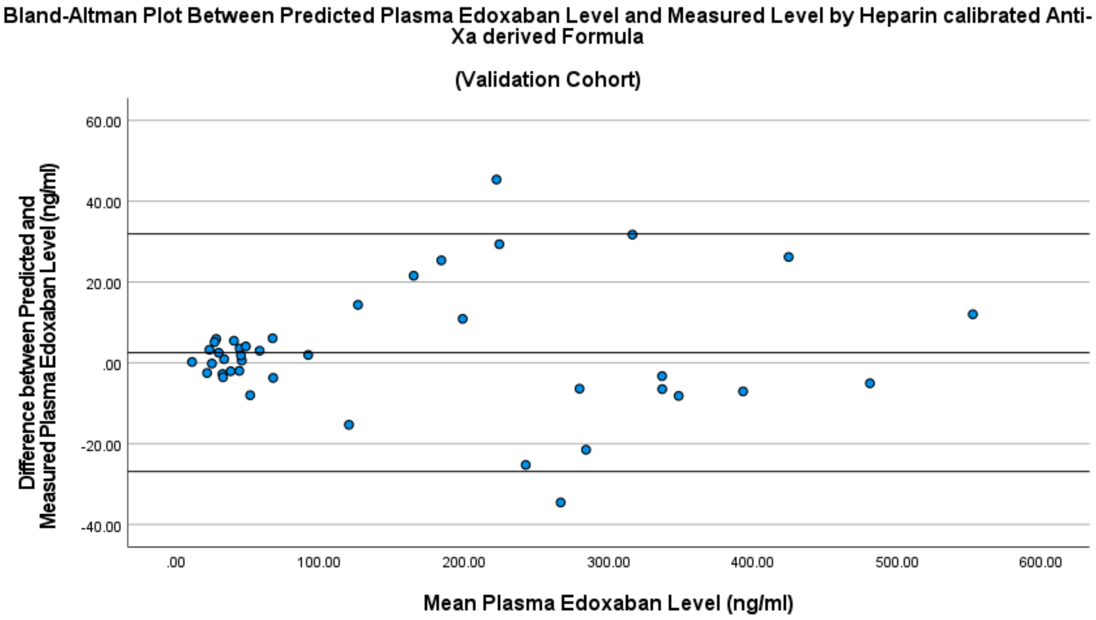

Supplementary Table S1: The diagnostic performance of using INR-derived formula to determine the cut-off plasma edoxaban level for intravenous thrombolytic therapy for patients present with ischaemic stroke (100 ng/ml). From the prediction formula, the INR of ≥ 1.2 would predict the plasma edoxaban level of 100 ng/ml. The results from A) Testing cohort showed sensitivity and specificity of 100% and 87.0% (95% C.I.: 73.2 – 100%) respectively and B) Validation cohort showed sensitivity and specificity of 100% and 86.4% (95% C.I.: 72.0 -100%) respectively.

A.

	Plasma edoxaban level > 100 ng/ml	Plasma edoxaban level ≤100 ng/ml	Total
INR ≥ 1.2	19	3	22
INR <1.2	0	20	20
Total	19	23	42

B.

	Plasma edoxaban level > 100 ng/ml	Plasma edoxaban level ≤100 ng/ml	Total
INR ≥ 1.2	19	3	22
INR <1.2	0	19	19
Total	19	22	41

Supplementary Table S2: The diagnostic performance of using INR-derived formula to determine the cut-off plasma edoxaban level for managing patients with bleeding complications or prior to urgent invasive procedures (50 ng/ml). From the prediction formula, the INR of ≥ 1.1 would predict the plasma edoxaban level of 50 ng/ml. The results from A) Testing cohort showed sensitivity and specificity of 100% and 65% (95% C.I.: 44.1 – 85.9%) respectively and B) Validation cohort showed sensitivity and specificity of 95.8% (95% C.I: 87.8 – 100%) and 58.8% (95% C.I.: 35.4 – 82.2%) respectively.

A.

	Plasma edoxaban level > 50 ng/ml	Plasma edoxaban level ≤ 50 ng/ml	Total
INR ≥ 1.1	22	7	29
INR <1.1	0	13	13
Total	22	20	42

B.

	Plasma edoxaban level > 50 ng/ml	Plasma edoxaban level ≤ 50 ng/ml	Total
INR ≥ 1.1	23	7	30
INR <1.1	1	10	11
Total	24	17	41

Supplementary Table S3: The diagnostic performance of using heparin calibrated anti-Xa activity derived formula to determine the cut-off plasma edoxaban level for administrating intravenous thrombolysis in patients presenting with ischaemic stroke (100 ng/ml). From the prediction formula, the anti-Xa activity of ≥ 0.64 would predict the plasma edoxaban level of 100 ng/ml. The results from A) Testing cohort showed 100% sensitivity and specificity and B) Validation cohort also showed 100% sensitivity and specificity.

A.

	Plasma edoxaban level > 100 ng/ml	Plasma edoxaban level ≤ 100 ng/ml	Total
Anti-Xa ≥ 0.64	19	0	19
Anti-Xa < 0.64	0	23	23
Total	19	23	42

B.

	Plasma edoxaban level > 100 ng/ml	Plasma edoxaban level ≤ 100 ng/ml	Total
Anti-Xa ≥ 0.64	19	0	19
Anti-Xa < 0.64	0	22	22
Total	19	22	41

Supplementary Table S4: The diagnostic performance of using heparin calibrated anti-Xa activity derived formula to determine the cut-off plasma edoxaban level for managing patients with bleeding complication or prior invasive procedures (50 ng/ml). From the prediction formula, the anti-Xa activity of ≥ 0.31 would predict the plasma edoxaban level of 50 ng/ml. The results from A) Testing cohort showed 100% sensitivity and specificity and B) Validation cohort showed sensitivity and specificity of 95.8% (95% C.I.: 87.8 – 100%) and 93.8% (95% C.I.: 81.9 – 100%) respectively.

A.

	Plasma edoxaban level > 50 ng/ml	Plasma edoxaban level ≤ 50 ng/ml	Total
Anti-Xa ≥ 0.31	22	0	22
Anti-Xa < 0.31	0	20	20
Total	22	20	42

B.

	Plasma edoxaban level > 50 ng/ml	Plasma edoxaban level ≤ 50 ng/ml	Total
Anti-Xa ≥ 0.31	23	1	24
Anti-Xa < 0.31	1	15	16
Total	24	16	40

Supplementary Table S5: Details of clinical indications for requesting plasma edoxaban level.

Patient Number	INR	Heparin calibrated anti-Xa activity	Plasma Edoxaban level (ng/ml)	Indications for requesting plasma edoxaban level
ER1	1.1	0.19	35.58	Acute-on-chronic renal failure
ER1 (same patient as above)	1.1	0.25	44.88	Acute-on-chronic renal failure
ER2	1.3	0.38	58.08	Prior to invasive procedure
ER2 (same patient as above)	1.3	0.30	54.64	Prior to invasive procedure
ER2 (same patient as above)	1.2	0.07	15.37	Prior to invasive procedure
ER2 (same patient as above)	1.2	0.40	62.55	Prior to invasive procedure
ER3	0.9	0.24	44.25	Potential drug interaction
ER4	1.1	0.05	18.35	Bleeding complications
ER5	1.2	0.05	8	Prior to invasive procedure
ER6	1.6	1.26	197.12	Potential drug interaction
ER7	1.5	0.72	107.42	Suspected overdose
ER8	1.2	0.06	16.41	Prior to

				invasive procedure
ER9	1.4	0.05	20.06	Prior to invasive procedure
ER9 (same patient as above)	1.3	0.05	8	Prior to invasive procedure
ER10	0.9	0.09	8.54	Prior to invasive procedure
ER11	1.2	0.57	78.44	Potential drug interaction
ER12	2.2	1.78	246.47	Bleeding complications
ER13	1.4	0.19	35.71	Prior to invasive procedure
ER14	1.3	0.08	21.34	Bleeding complications
ER15	1.5	0.88	173.04	Prior to invasive procedure
ER15 (same patient as above)	1.5	0.85	170.75	Bleeding complications
ER15 (same patient as above)	1.3	0.46	92.02	Prior to invasive procedure
ER16	1.3	0.1	30.57	Prior to invasive procedure
ER17	1.1	0.05	8	Ischaemic stroke indicated for intravenous thrombolysis
ER18	1.0	0.06	16.26	Prior to

				invasive procedure
ER19	1.5	1.04	183.3	Suspected drug interaction
ER20	1.1	0.05	10.94	Extreme body weight

Supplementary Table S6: The diagnostic performance of using INR-derived formula to determine the cut-off plasma edoxaban level for administrating intravenous thrombolysis in patients presenting with ischaemic stroke (100 ng/ml). The data was generated from real-life clinical requests of plasma edoxaban level. From the prediction formula, the INR value of ≥ 1.2 would predict the plasma edoxaban level of 100 ng/ml. The sensitivity and specificity were 100% and 38.1% (95% C.I.: 17.3 – 58.9%) respectively.

	Plasma edoxaban level > 100 ng/ml	Plasma edoxaban level ≤ 100 ng/ml	Total
INR ≥ 1.2	6	13	19
INR <1.2	0	8	8
Total	6	21	27

Supplementary Table S7: The diagnostic performance of using INR-derived formula to determine the cut-off plasma edoxaban level for managing patients with bleeding complications or prior to urgent invasive procedures (50 ng/ml). The data was generated from real-life clinical requests of patients. From the prediction formula, the INR of ≥ 1.1 would predict the plasma edoxaban level of 50 ng/ml. The sensitivity was 100%. However, the specificity was rather low (18.8%, 95% C.I: 0 – 37.9%).

	Plasma edoxaban level > 50 ng/ml	Plasma edoxaban level ≤ 50 ng/ml	Total
INR ≥ 1.1	11	13	24
INR <1.1	0	3	3
Total	11	16	27
